# Blood indicators for short-term mortality risk in older patients with hip fracture: association and predictive value

**DOI:** 10.3389/fmed.2025.1684738

**Published:** 2025-11-27

**Authors:** Qing Zhou, Desheng Zhang, Yuxuan Wu, Xi Chen, Zhicong Wang

**Affiliations:** Department of Orthopedics, Deyang People’s Hospital, Deyang, Sichuan, China

**Keywords:** hip fracture, mortality, blood indicator, predictive value, older adult

## Abstract

**Objectives:**

To investigate blood indicators associated with short-term mortality risk in older patients with hip fracture, and further evaluate the incremental predictive value of incorporating these indicators into existing clinical models.

**Methods:**

Data from 1881 patients in our institutional hip fracture database between January 2013 and December 2023 were retrospectively analyzed. The study outcome was all-cause mortality within 90 days of admission. Stepwise logistic regression, the Boruta algorithm, and Lasso regression were performed to identify features associated with mortality risk. Following feature selection, two predictive models were developed: Model A (clinical indicators only) and Model B (both clinical and blood indicators). Predictive performance was assessed using the area under the curve (AUC), net reclassification improvement (NRI), and integrated discrimination improvement (IDI).

**Results:**

Of the 1881 patients, 217 (11.5%) died within 90 days. Stepwise logistic regression identified 12 significant features associated with mortality risk, the Boruta algorithm identified 25 important features, and Lasso regression analysis selected 18 features with non-zero coefficients (all *P* < 0.05). Model B significantly outperformed Model A across all feature selection methods (all *P* < 0.001): stepwise logistic regression (AUC: 0.822 vs. 0.753), the Boruta algorithm (AUC: 0.820 vs. 0.749), and Lasso regression (AUC: 0.826 vs. 0.756). Model B also showed significant reclassification improvements (all *P* < 0.001): stepwise logistic regression (NRI: 0.733, IDI: 0.107), the Boruta algorithm (NRI: 0.762, IDI: 0.113), and Lasso regression (NRI: 0.725, IDI: 0.112).

**Conclusion:**

Various blood indicators were associated with 90-day mortality in older patients with hip fracture, and significantly enhanced the predictive ability of clinical models for short-term mortality risk. By utilizing these blood indicators, clinicians can comprehensively and objectively assess the physiological status of hip fracture patients at admission, thereby facilitating the early identification of high-risk patients and guiding personalized treatment strategies.

## 1 Introduction

With aging intensifying, hip fractures have become a major global public health issue (1). Data from the Global Burden of Disease Study (GBD) revealed a rapid rise in hip fracture incidence among people aged 60 years and older across 204 countries and territories from 1990 to 2019 (1). Another study projected a 1.91-fold increase in annual hip fractures across 19 countries and regions by 2050 compared to 2018 (2). Notably, despite significant medical advancements, the mortality rate among older patients with hip fracture remains high. Recently, a meta-analysis of 244 studies involving over a million hip fracture patients reported that in-hospital, 30-day, and 1-year mortality rates were 1.4–5.5%, 1.2–7.4%, and 10.8–23.8%, respectively (3). As is well known, identifying high-risk individuals early and accurately is crucial for the timely initiation and optimization of personalized interventions, thereby improving the prognosis of hip fracture patients (4).

To accurately predict the mortality risk in patients sustaining hip fractures, numerous risk prediction models have been established. Among these, some are specifically designed for hip fractures, including the Nottingham Hip Fracture Score (NHFS) (5), the Almelo Hip Fracture Score (AHFS) (6), and the models developed by Jiang et al. (7) and Holt et al. (8). Other models, such as the Charlson Comorbidity Index (CCI) (9), the American Society of Anesthesiologists (ASA) classification (10), and the American College of Surgeons National Surgical Quality Improvement Program (ACS-NSQIP) Surgical Risk Calculator (11), are also used but are not tailored for hip fractures. However, these models may be limited by complexity, inherent subjectivity and potential overestimation of mortality risk (12–14).

In clinical practice, routine blood indicators are easily accessible and cost-effective, and can objectively reflect the severity of a patient’s condition. Many blood indicators have been demonstrated to be significantly associated with the mortality risk in hip fracture patients, such as hemoglobin (15), lymphocyte count (16), neutrophil count (17), and albumin level (18). A recent systematic review indicated that six of 23 mortality prediction models were commonly used for hip fracture patients, including the NHFS, CCI, AHFS, Jiang et al., Holt et al., and ACS-NSQIP (19). However, only two of these six models (NHFS and AHFS) incorporate a single blood parameter, specifically hemoglobin. Therefore, this study aimed to systematically investigate blood indicators associated with short-term mortality risk in older patients with hip fracture, and further evaluate the incremental predictive value of incorporating these indicators into existing clinical models.

## 2 Materials and methods

### 2.1 Patients and study design

**FIGURE 1:**
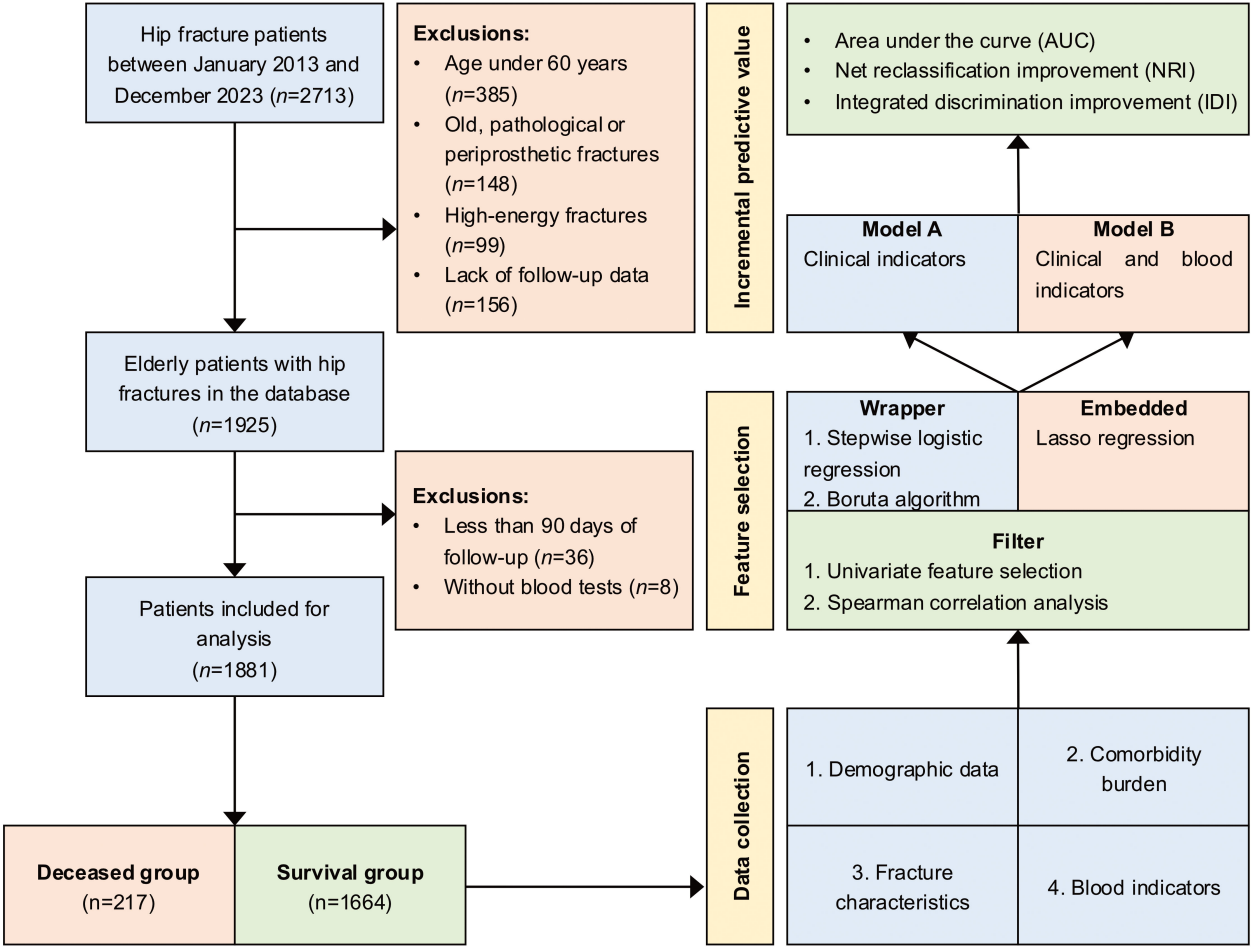
Study flowchart. This flowchart illustrates the study design, including case inclusion and exclusion criteria, data collection process, feature selection methods, and evaluation of incremental predictive value.

This study retrospectively analyzed data from our institutional hip fracture database, which has been extensively used for prognostic research in hip fractures (20–23). Between January 2013 and December 2023, a total of 2713 patients diagnosed with femoral neck or intertrochanteric fractures were initially enrolled in the database. Patients were excluded from the database if they met any of the following criteria: (1) aged under 60 years; (2) old fractures (> 3 weeks after injury), pathological fractures, or periprosthetic fractures; (3) high-energy fractures (e.g., traffic accidents, falls from a height greater than standing height); (4) lack of follow-up data. In this study, patients with less than 90 days of follow-up or without blood tests were further excluded. The flowchart of patient selection is illustrated in Figure 1. Our Institutional Ethics Committee approved the study protocol (No. 2022-04-040-K01), and patients provided written informed consent for the research use of their clinical data.

### 2.2 Data collection

Previous study has reported that when the missing data rate exceeds 15.0%, the multiple imputation by chained equations (MICE) method may yield unreliable results (24). Therefore, the following variables were extracted from the database, and those with missing rates over 15.0% were excluded to ensure data quality and the reliability of subsequent analyses.

(1) Demographic data included age, sex, body mass index (BMI), smoking status, and marital status. According to our previous study (22), smoking status was categorized as non-smoker or smoker (current or former), and marital status was classified as widowed or other (married, divorced or never-married).

(2) For objective assessment of patients’ comorbidity burden, the CCI was calculated for each patient based on admission diagnoses and past medical history.

(3) Fracture characteristics included time to admission (TTA) and fracture type. TTA, defined as the interval from injury occurrence to hospital admission, was determined from admission records (25). Fracture type was grouped as either femoral neck or intertrochanteric fracture based on discharge diagnoses and imaging findings.

(4) Given the documented association between blood indicators and mortality in hip fracture patients, the following blood indicators were collected at the first 24 h after admission.

(i) Blood routine tests included white blood cell (WBC), neutrophil, lymphocyte, monocyte, red blood cell (RBC), hemoglobin, hematocrit, mean corpuscular volume (MCV), mean corpuscular hemoglobin (MCH), mean corpuscular hemoglobin concentration (MCHC), red cell distribution width-standard deviation (RDW-SD), red cell distribution width-coefficient of variation (RDW-CV), and platelet.

(ii) Biochemical tests included liver function (albumin, prealbumin, alanine aminotransferase [ALT], alkaline phosphatase [ALP]), renal function (creatinine, blood urea nitrogen [BUN], uric acid), electrolytes (potassium, sodium, calcium), and blood glucose.

(iii) Coagulation function tests included international normalized ratio (INR), prothrombin time (PT), and activated partial thromboplastin time (APTT).

(5) Other blood indicators that have not been previously reported to be associated with mortality in hip fracture patients were also collected. These included eosinophil, basophil, mean platelet volume (MPV), plateletcrit, fibrinogen, thrombin time, antithrombin III activity (AT-III), β2-microglobulin (β2-MG), adenosine deaminase (ADA), aspartate aminotransferase (AST), total bile acids (TBA), direct bilirubin (D-BIL), total bilirubin (T-BIL), globulin, total protein, albumin to globulin ratio (AGR), magnesium (Mg), phosphorus (P), chloride (CL), and bicarbonate (HCO_3_).

### 2.3 Study outcome

The study outcome was the short-term mortality rate, defined as all-cause mortality within 90 days of admission (26). Patient survival status was ascertained through three methods (22). (1) For in-hospital deaths, the exact time of death was obtained from hospital death records. (2) For discharged patients, annual telephone follow-up was conducted until May 2024, and the date of death was recorded when applicable. (3) If telephone follow-up failed after three attempts, post-discharge survival status was verified through outpatient, emergency or inpatient medical records. Cases for whom survival status could not be determined using these three methods were considered lost to follow-up, resulting in a loss to follow-up rate of 7.50% in our database.

### 2.4 Statistical analysis

Prior to analysis, missing values were detected in 28 variables, with missing data rates ranging from 0.3 to 13.8%. Missing values were imputed using the random forest method for multiple imputation (27). Continuous variables are expressed as mean ± standard deviation (SD) or median [interquartile range (IQR)] based on the Shapiro-Wilk normality test results, and categorical variables are presented as numbers (percentages). Differences between the survival and deceased groups were compared using the *t*-test, Wilcoxon rank-sum test or Pearson’s chi-square test.

To identify variables associated with mortality risk, this study employed three feature selection approaches: filter, wrapper and embedded methods (Figure 1). Initially, univariate feature selection was performed to eliminate features that were not significantly associated with the outcome (*P* > 0.05), and Spearman correlation analysis was used to remove redundant features with strong correlation (|ρ| ≥ 0.7). Subsequently, stepwise logistic regression was employed to identify features associated with mortality, with mortality risk quantified as odds ratio (OR) and 95% confidence interval (*CI*). Additionally, the Boruta algorithm, a feature selection method based on random forests, was applied to assess feature importance through the use of shadow features and iterative processes (28). Lasso regression, a widely used embedded feature selection method, was also applied (29). To optimize the selection of features associated with mortality, the lambda value corresponding to the minimum mean squared error (lambda.min) was chosen as the regularization parameter.

Following feature selection, logistic regression was conducted to construct two predictive models: Model A, which included only clinical indicators, and Model B, which incorporated both clinical and blood indicators (Figure 1). The area under the curve (AUC), net reclassification improvement (NRI), and integrated discrimination improvement (IDI) were used to evaluate the incremental predictive value of incorporating additional indicators into the existing model (30). Receiver operating characteristic (ROC) curves were plotted to calculate the AUC, and the DeLong test was employed to compare the AUC differences between the two models. The “PredictABEL” package was utilized to compute and compare the NRI and IDI for both models.

All reported *P-*values were two-sided, and *P* < 0.05 was considered statistically significance. Statistical analyses were performed using R statistical software (version 4.2.2; R Project for Statistical Computing).

## 3 Results

### 3.1 Patient characteristics

A total of 1,881 older patients with hip fracture were included in this study, of whom 35.5% were male, with a median age of 80.0 years. The most common comorbidities were chronic pulmonary disease (21.6%), diabetes mellitus (21.4%), and cerebrovascular disease (12.7%). The characteristics of the patients are presented in Table 1. During the 90-day follow-up period, 217 deaths occurred, yielding a 90-day mortality rate of 11.5% (95% *CI*: 10.2–13.1%). Among these, 39 deaths (2.1%) were in-hospital, and 113 deaths (6.0%) occurred within 30 days of admission. Compared with the survival group, the deceased group was older, had a greater proportion of males and smokers, a higher CCI score, longer TTA, and lower BMI (all *P* < 0.05). However, marital status and fracture type approached but did not reach statistical significance (*P* > 0.05). Among the 47 blood indicators, 11 showed no statistically significant differences between groups, whereas the remaining indicators exhibited statistically significant differences.

**TABLE 1:** Comparison of patient characteristics between the survival and deceased groups.

Variables	All (*n* = 1881)	Survival group (*n* = 1664)	Deceased group (*n* = 217)	*P-*value
Age, years	80.0 (73.0, 85.0)	79.0 (72.0, 85.0)	84.0 (78.0, 87.0)	** < 0.001**
Male, *n* (%)	668 (35.5)	568 (34.1)	100 (46.1)	** < 0.001**
BMI, Kg/m^2^	21.3 (19.1, 23.9)	21.4 (19.1, 23.9)	20.8 (18.7, 22.6)	**0.003**
Smoker, *n* (%)	436 (23.2)	369 (22.2)	67 (30.9)	**0.006**
Widowed, *n* (%)	507 (27.0)	436 (26.2)	71 (32.7)	0.051
CCI	1.0 (0, 1.0)	1.0 (0, 1.0)	1.0 (1.0, 3.0)	** < 0.001**
TTA, hours	10.0 (3.0, 48.0)	9.0 (3.0, 48.0)	24.0 (5.0, 72.0)	**0.001**
Fracture type, *n*(%)				0.091
Femoral neck	899 (47.8)	807 (48.5)	92 (42.4)	
Intertrochanteric	982 (52.2)	857 (51.5)	125 (57.6)	
**Blood indicators**				
WBC, 10^9^/L	8.8 (6.8, 11.1)	8.7 (6.8, 10.9)	9.5 (6.6, 12.0)	**0.043**
Neutrophil, 10^9^/L	7.1 (5.2, 9.5)	7.0 (5.2, 9.4)	7.8 (5.2, 10.7)	**0.018**
Lymphocyte, 10^9^/L	0.9 (0.6, 1.2)	0.9 (0.6, 1.2)	0.7 (0.5, 0.9)	** < 0.001**
Monocyte, 10^9^/L	0.5 (0.4, 0.7)	0.5 (0.4, 0.7)	0.6 (0.4, 0.8)	**0.027**
RBC, 10^9^/L	3.7 (3.3, 4.2)	3.8 (3.3, 4.2)	3.5 (2.9, 4.0)	** < 0.001**
Hemoglobin, g/L	112.0 (98.0, 125.0)	113.0 (99.0, 126.0)	103.0 (86.0, 121.0)	** < 0.001**
Hematocrit,%	34.7 (30.4, 38.5)	35.0 (31.1, 38.7)	31.9 (27.4, 36.9)	** < 0.001**
MCV, fL	93.6 (90.0, 97.3)	93.5 (90.0, 97.2)	94.1 (88.9, 97.4)	0.791
MCH, pg	30.4 (28.9, 31.7)	30.4 (29.0, 31.7)	30.4 (28.6, 31.6)	0.373
MCHC, g/L	323.0 (315.0, 331.0)	324.0 (316.0, 332.0)	322.0 (313.0, 330.0)	**0.026**
RDW-SD, fL	46.8 (44.1, 50.3)	46.7 (44.0, 49.8)	48.7 (45.5, 52.8)	** < 0.001**
RDW-CV,%	13.8 (13.2, 14.7)	13.8 (13.1, 14.6)	14.4 (13.6, 15.6)	** < 0.001**
Platelet, 10^9^/L	147.0 (108.0, 194.0)	147.0 (108.0, 194.0)	149.0 (108.0, 200.0)	0.854
Albumin, g/L	38.9 (35.8, 41.9)	39.2 (36.2, 42.1)	36.5 (33.3, 39.3)	** < 0.001**
Prealbumin, mg/L	171.0 (130.9, 210.0)	174.8 (135.2, 213.1)	145.0 (111.0, 183.2)	** < 0.001**
ALT, U/L	16.0 (12.0, 23.0)	16.0 (12.0, 23.0)	16.0 (12.0, 23.0)	0.604
ALP, U/L	84.0 (68.0, 103.0)	83.0 (68.0, 102.0)	89.0 (68.0, 117.0)	**0.013**
Creatinine, μmol/L	67.0 (55.0, 87.0)	65.0 (55.0, 83.3)	86.3 (62.0, 128.6)	** < 0.001**
BUN, mmol/L	6.8 (5.3, 9.0)	6.6 (5.2, 8.6)	9.3 (6.8, 12.7)	** < 0.001**
Uric acid, μmol/L	284.0 (219.4, 367.0)	279.0 (215.9, 360.0)	315.7 (260.0, 433.3)	** < 0.001**
Potassium, mmol/L	3.9 (3.6, 4.2)	3.9 (3.6, 4.2)	4.0 (3.6, 4.4)	**0.004**
Sodium, mmol/L	141.5 (139.1, 143.7)	141.6 (139.3, 143.7)	140.5 (137.6, 143.1)	** < 0.001**
Calcium, mmol/L	2.2 (2.0, 2.3)	2.2 (2.1, 2.3)	2.1 (2.0, 2.2)	** < 0.001**
Glucose, mmol/L	7.0 (5.9, 8.9)	6.9 (5.8, 8.8)	7.6 (6.1, 10.3)	** < 0.001**
INR	1.0 (1.0, 1.1)	1.0 (1.0, 1.1)	1.1 (1.0, 1.2)	** < 0.001**
PT, s	12.1 (11.5, 12.8)	12.0 (11.5, 12.7)	12.6 (11.7, 13.5)	** < 0.001**
APTT, s	27.7 (25.2, 30.9)	27.6 (25.1, 30.6)	28.9 (26.3, 32.7)	** < 0.001**
Eosinophil, 10^9^/L	0.03 (0.01, 0.10)	0.03 (0.01, 0.10)	0.03 (0, 0.10)	0.100
Basophil, 10^9^/L	0.02 (0.01, 0.03)	0.02 (0.01, 0.03)	0.02 (0.01, 0.03)	0.347
MPV, fL	12.0 (11.0, 13.0)	12.0 (11.0, 13.0)	11.8 (10.9, 12.9)	**0.023**
Plateletcrit	0.2 (0.1, 0.2)	0.2 (0.1, 0.2)	0.2 (0.1, 0.2)	0.524
Fibrinogen, g/L	3.3 (2.6, 4.1)	3.2 (2.6, 4.1)	3.5 (2.7, 4.5)	**0.006**
Thrombin time, s	17.4 (16.3, 18.6)	17.4 (16.2, 18.6)	17.4 (16.4, 18.7)	0.749
AT-III,%	86.5 (77.3, 96)	86.7 (78.1, 96.3)	83.2 (71.1, 92.9)	** < 0.001**
β2-MG, mg/L	2.7 (2.1, 3.7)	2.6 (2.0, 3.5)	3.8 (2.7, 6.3)	** < 0.001**
ADA, U/L	11.6 (8.7, 15.4)	11.0 (8.6, 15.0)	13.1 (9.7, 18.6)	** < 0.001**
AST, U/L	23.0 (19.0, 29.0)	23.0 (19.0, 28.0)	25.0 (19.0, 34.0)	**0.016**
TBA, μmol/L	5.0 (2.9, 8.7)	4.9 (2.9, 8.6)	5.4 (3.3, 10.2)	**0.040**
D-BIL, μmol/L	4.5 (3.0, 6.8)	4.4 (3.0, 6.6)	5.1 (3.3, 8.8)	** < 0.001**
T-BIL, μmol/L	14.7 (10.5, 20.2)	14.7 (10.6, 20.1)	15.2 (9.8, 21.8)	0.508
Globulin, g/L	25.8 (22.7, 29.5)	25.7 (22.6, 29.4)	27.0 (23.2, 30.7)	**0.017**
Total protein, g/L	65.2 ± 7.2	65.4 ± 7.1	63.7 ± 7.4	**0.002**
AGR	1.5 (1.3, 1.7)	1.5 (1.3, 1.7)	1.4 (1.1, 1.6)	** < 0.001**
Mg, mmol/L	0.9 (0.8, 0.9)	0.9 (0.8, 0.9)	0.9 (0.8, 0.9)	0.336
P, mmol/L	0.9 (0.8, 1.1)	0.9 (0.8, 1.1)	1.0 (0.9, 1.2)	** < 0.001**
Chloride, mmol/L	104.1 (101.1, 106.9)	104.1 (101.2, 106.8)	103.4 (99.9, 107.2)	0.090
HCO3, mmol/L	24.1 (21.7, 26.6)	24.1 (21.8, 26.6)	23.4 (20.5, 26.6)	**0.023**

Continuous variables are expressed as mean ± SD or median (IQR), and categorical variables are presented as numbers (%). Bold *P-*values denote significance.

### 3.2 Feature selection

**FIGURE 2:**
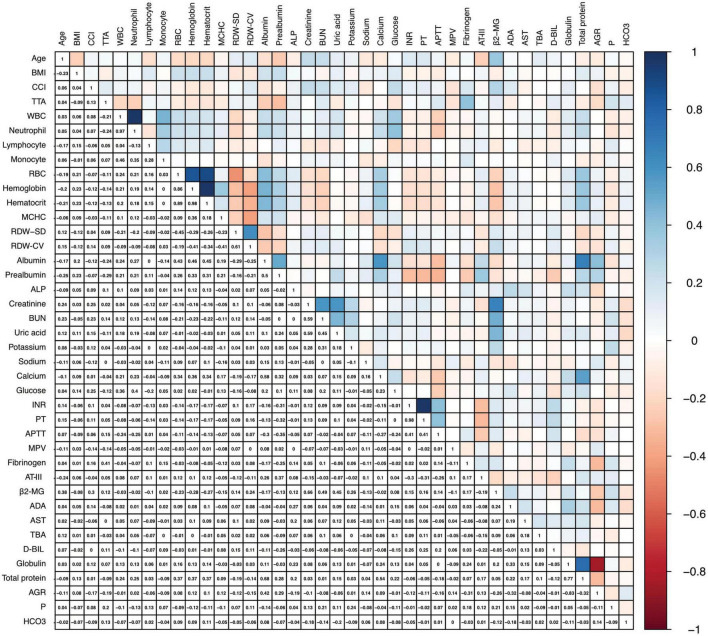
Spearman correlation heatmap of predictive features. Blue shading indicates positive correlations, while red shading indicates negative correlations.

After excluding 13 statistically non-significant variables (Table 1), Spearman correlation analysis was conducted on the remaining features. As shown in Figure 2, seven pairs of features exhibited strong correlations: WBC and neutrophil (ρ = 0.973), RBC and hemoglobin (ρ = 0.858), RBC and hematocrit (ρ = 0.893), hemoglobin and hematocrit (ρ = 0.976), INR and PT (ρ = 0.976), globulin and total protein (ρ = 0.767), globulin and AGR (ρ = −0.830). Accordingly, the five redundant features (neutrophil, RBC, hematocrit, INR and globulin) were excluded from further analysis.

**FIGURE 3:**
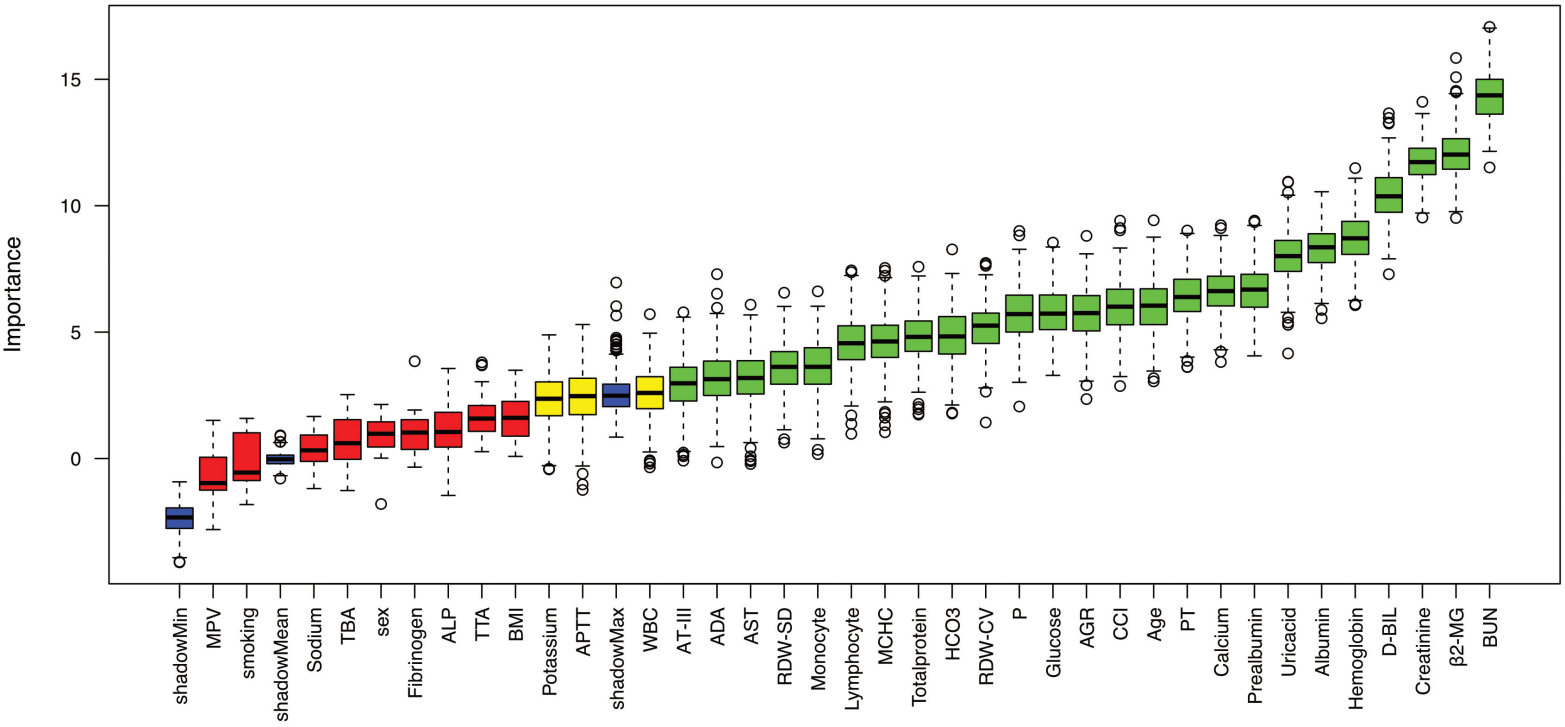
Feature selection results using the Boruta algorithm. Features are categorized based on their importance: important features (green), possibly important features (yellow), unimportant features (red), and shadow features (blue).

**FIGURE 4:**
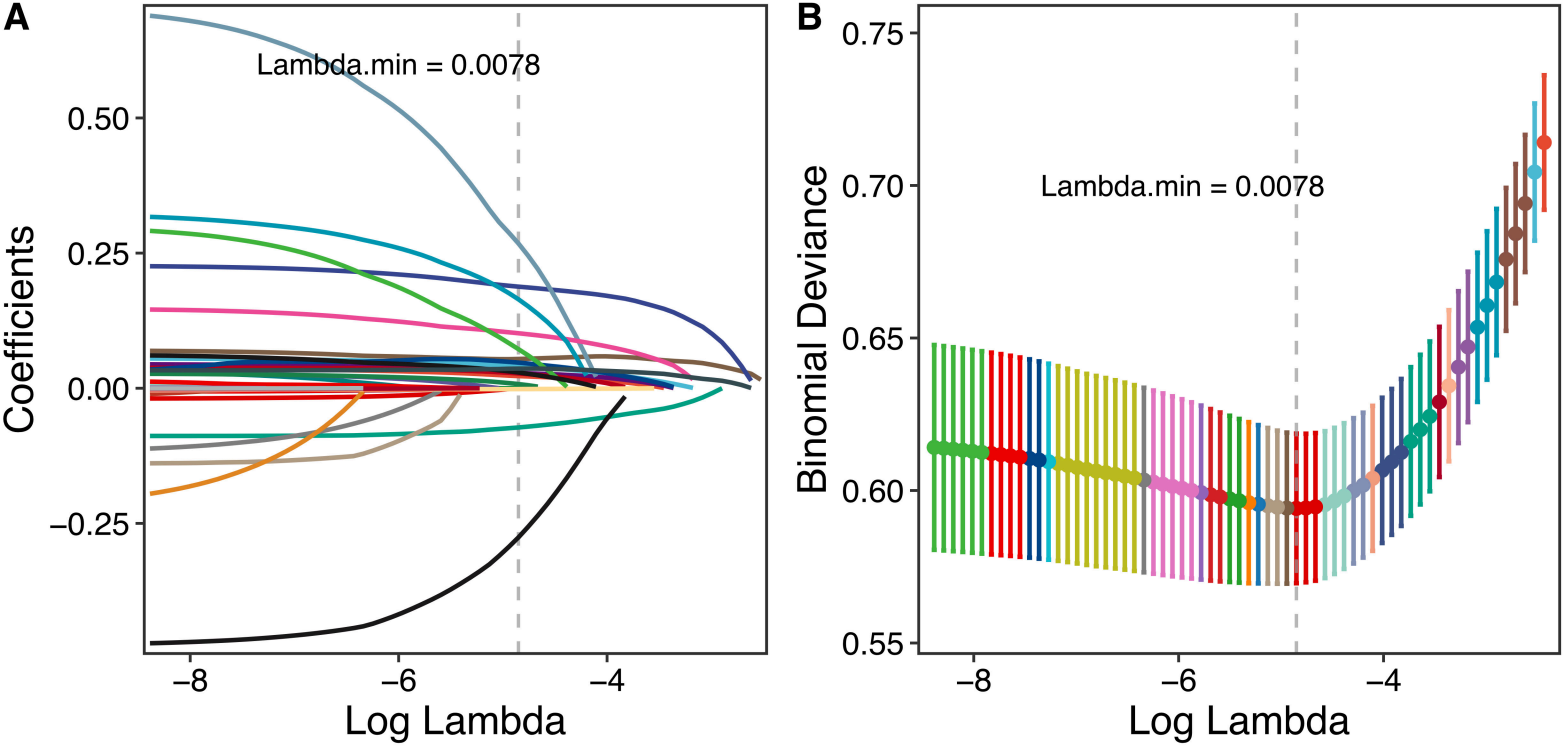
Lasso regression results. **(A)** The coefficient plot of the Lasso regression model, illustrating the trajectory of each predictor’s coefficient as the regularization parameter (lambda) increases. **(B)** The selection of the optimal lambda value through cross-validation, highlighting the point at which the model achieves the best balance between bias and variance, ensuring robust predictor selection.

Subsequently, stepwise logistic regression identified 12 features significantly associated with mortality risk, including age, smoking status, CCI, WBC, lymphocyte, albumin, BUN, glucose, PT, β2-MG, ADA, and D-BIL (Table 2). Concurrently, the Boruta algorithm identified 25 important features, which were ranked in descending order of importance as follows: BUN, β2-MG, creatinine, D-BIL, hemoglobin, albumin, uric acid, prealbumin, calcium, PT, age, CCI, AGR, glucose, P, RDW-CV, HCO3, total protein, MCHC, lymphocyte, monocyte, RDW-SD, AST, ADA, and AT-III (Figure 3). Lasso regression analysis demonstrated that as the regularization parameter lambda increased, the regression coefficients were progressively compressed, with some eventually reaching zero (Figure 4A). The optimal lambda value, corresponding to the minimum mean squared error, was determined to be 0.0078 through five-fold cross-validation (Figure 4B). Consequently, 18 features with non-zero coefficients were selected, including age, sex, smoking, CCI, WBC, lymphocyte, RDW-CV, albumin, prealbumin, creatinine, BUN, glucose, PT, MPV, β2-MG, ADA, D-BIL, and P.

**TABLE 2:** Feature selection for independent mortality predictors using stepwise logistic regression.

Variables	Univariate		Multivariate	
	OR (95% CI)	*P-*value	OR (95% CI)	*P-*value
Age	1.063 (1.044–1.083)	< 0.001	1.060 (1.037–1.083)	< 0.001
Smoker	1.568 (1.144–2.13)	0.005	1.479 (1.039–2.088)	0.028
CCI	1.491 (1.376–1.618)	< 0.001	1.230 (1.107−1.364)	< 0.001
WBC	1.045 (1.006–1.085)	0.021	1.054 (1.010–1.099)	0.015
Lymphocyte	0.383 (0.26–0.553)	< 0.001	0.583 (0.392–0.834)	0.005
Albumin	0.877 (0.849–0.906)	< 0.001	0.895 (0.863–0.928)	< 0.001
BUN	1.143 (1.113–1.174)	< 0.001	1.069 (1.033–1.107)	< 0.001
Glucose	1.064 (1.034–1.095)	< 0.001	1.037 (1.000–1.074)	0.044
PT	1.307 (1.203–1.428)	< 0.001	1.151 (1.045–1.262)	0.003
β2-MG	1.156 (1.118–1.198)	< 0.001	1.042 (1.000−1.086)	0.049
ADA	1.048 (1.03−1.066)	< 0.001	1.035 (1.013−1.057)	0.001
D-BIL	1.071 (1.041−1.103)	< 0.001	1.046 (1.013−1.080)	0.005

### 3.3 Incremental predictive value of blood indicators

**FIGURE 5:**
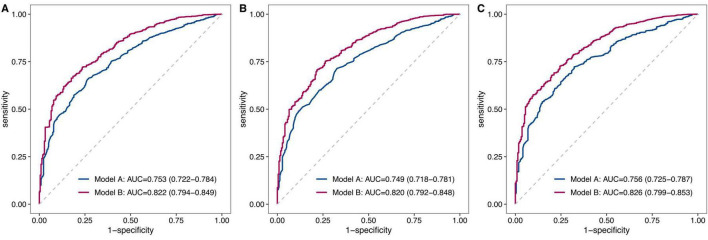
Comparative analysis of ROC curves between model A and model B. This figure presents a comparative analysis of the ROC curves for two predictive models: Model A, which includes only clinical indicators, and Model B, which incorporates both clinical and blood indicators. The analysis was conducted using three feature selection methods: stepwise logistic regression **(A)**, the Boruta algorithm **(B)**, and Lasso regression **(C)**. In each figure, the red curve represents Model B, while the blue curve represents Model A. The AUC values were provided for each model, illustrating the discriminative ability of each model in predicting 90-day mortality.

Following feature selection, two predictive models were developed: Model A (clinical indicators only) and Model B (both clinical and blood indicators, Table 3). As evaluated by ROC curve analysis, Model B exhibited significantly superior performance, with higher AUC values compared to Model A, regardless of whether blood indicators were selected using stepwise logistic regression (0.822 [0.794–0.849] vs. 0.753 [0.722–0.784], *P* < 0.001, Figure 5A), the Boruta algorithm (0.820 [0.792–0.848] vs. 0.749 [0.718–0.781], *P* < 0.001, Figure 5B), or Lasso regression (0.826 [0.799–0.853] vs. 0.756 [0.725–0.787], *P* < 0.001, Figure 5C). Furthermore, Model B significantly enhanced reclassification ability after incorporating blood indicators selected by three different methods: stepwise logistic regression (NRI: 0.733 [0.597–0.868], IDI: 0.107 [0.082–0.133]), the Boruta algorithm (NRI: 0.762 [0.629–0.895], IDI: 0.113 [0.086–0.140]), and Lasso regression (NRI: 0.725 [0.589–0.860], IDI: 0.112 [0.085–0.139]) (all *P* < 0.001, Table 3).

**TABLE 3:** Comparison of NRI and IDI differences between model A (clinical indicators only) and model B (clinical and blood indicators) for predicting 90-day mortality.

Feature selection methods	Feature subsets	NRI (95% CI)	IDI (95% CI)
Stepwise logistic regression	Model A: age, smoking, CCI Model B: Model A and WBC, lymphocyte, albumin, BUN, glucose, PT, β2-MG, ADA, D-BIL.	0.733 (0.597–0.868)	0.107 (0.082–0.133)
Boruta algorithm	Model A: age, CCI Model B: Model A and BUN, β2-MG, creatinine, D-BIL, hemoglobin, albumin, uric acid, prealbumin, calcium, PT, AGR, glucose, P, RDW-CV, HCO3, total protein, MCHC, lymphocyte, monocyte, RDW-SD, AST, ADA, AT-III.	0.762 (0.629–0.895)	0.113 (0.086–0.140)
Lasso regression	Model A: age, sex, smoking, CCI Model B: Model A and WBC, lymphocyte, RDW-CV, albumin, prealbumin, creatinine, BUN, glucose, PT, MPV, β2-MG, ADA, D-BIL, P.	0.725 (0.589–0.860)	0.112 (0.085–0.139)

## 4 Discussion

Hip fractures in the older adults are associated with high mortality rates, and numerous risk factors for short-term mortality have been reported in previous studies (4). This study systematically analyzed blood indicators available at the time of admission, and revealed significant associations between 25 blood indicators and 90-day mortality. Among these, eight blood indicators were consistently identified by stepwise logistic regression, the Boruta algorithm and Lasso regression, including lymphocyte, albumin, BUN, glucose, PT, β2-MG, ADA, and D-BIL. Four indicators were selected by two of the three methods: WBC, RDW-CV, prealbumin, and creatinine. By utilizing these blood indicators, clinicians can comprehensively and objectively evaluate the physiological status of hip fracture patients, including inflammation and immune function (WBC, lymphocyte, ADA), liver function (albumin, prealbumin, D-BIL), kidney function (BUN, creatinine, β2-MG), glucose metabolism (glucose), and coagulation function (PT).

Hip fracture trauma induces an acute inflammatory response, and age-related immune senescence may contribute to immune dysfunction and dysregulated inflammation in older patients (31, 32). Elevated WBC counts indicate a systemic inflammatory response that can lead to tissue damage and complications, whereas low lymphocyte counts suggest immunosuppression, which impairs the body’s ability to fight infections and promote healing. Previous studies have reported that WBC counts above the normal range were associated with a 1.139-fold increased risk of 30-day mortality in hip fracture patients (33), and a lymphocyte count < 1 × 10^9^/L was associated with a 1.728-fold increased risk of in-hospital mortality (16). ADA is a key enzyme in purine nucleoside metabolism, catalyzing the deamination of adenosine and deoxyadenosine, thereby attenuating the immunosuppressive effects of adenosine and playing a critical role in maintaining immune homeostasis (34). As shown in Table 2, each 1.0 U/L increment in serum ADA activity was associated with a 3.5% increase in the risk of 90-day mortality. To date, no studies have reported an association between ADA levels and mortality risk in hip fracture patients. However, ADA has been identified as a prognostic biomarker for severe fever with thrombocytopenia syndrome (35). Moreover, our previous studies have revealed that immune-inflammatory indicators, including the platelet-to-lymphocyte ratio (PLR) (20), systemic immune-inflammation index (SII) (21), systemic inflammation response index (SIRI) (22), and hemoglobin, albumin, lymphocyte and platelet (HALP) (23), were significantly associated with both short- and long-term mortality in hip fracture patients. It should be noted that our database did not include information regarding the presence of infections at the time of admission. Therefore, additional studies are needed to confirm the relationship between inflammatory markers and increased mortality risk in hip fracture patients.

Malnutrition is highly prevalent among older patients with hip fracture, with a systematic review reporting incidence rates of up to 39.4% (36). Serum ALB and prealbumin are widely used biomarkers for nutritional assessment, reflecting both nutritional status and hepatic protein synthesis capacity. Hypoalbuminemia is frequently associated with malnutrition, chronic inflammation, and liver dysfunction, all of which can adversely affect wound healing, immune function, and overall recovery, thereby increasing the risk of complications and poor outcomes in hip fracture patients. This study showed that each 1.0 g/L increase in ALB was associated with a 10.5% reduction in the risk of 90-day mortality. When ALB levels were dichotomized at a threshold of 35.0 g/L, patients with hypoalbuminemia exhibited a 1.78-fold higher mortality risk (37). Prealbumin, with its shorter half-life, more rapidly reflects short-term changes in nutritional status than other proteins such as albumin. Chen et al. (38) reported that prealbumin levels were independently associated with long-term mortality risk in 2,387 hip fracture patients with a mean follow-up of 37.6 months. In addition, D-BIL is a well-established biomarker for evaluating hepatic function, reflecting the liver’s ability for bilirubin metabolism. Elevated D-BIL levels typically signify impaired bilirubin conjugation and excretion, which are hallmarks of substantial liver dysfunction. In this study, we observed that each 1.0 μmol/L increase in D-BIL was associated with a 4.6% increase in the risk of 90-day mortality. Although no previous studies have specifically investigated the relationship between D-BIL levels and mortality risk in hip fracture patients, elevated D-BIL levels have been significantly associated with poor prognosis in other diseases, such as heart failure (39), and ischemic stroke (40).

In older adults, chronic kidney disease (CKD) disrupts bone and mineral metabolism and alters vitamin D metabolism, thereby exacerbating osteoporosis and increasing the risk of hip fractures (41). Meanwhile, impaired kidney function can lead to electrolyte imbalances and metabolic complications, which can further complicate recovery and increase mortality risk. Patients with CKD who experience a hip fracture faced a 2.17-fold higher mortality risk compared to those without CKD. This risk further increased to 6.21-fold in patients receiving peritoneal dialysis and 3.62-fold in those on hemodialysis (42). In clinical practice, BUN and creatinine are widely used biomarkers for assessing renal function. When BUN levels were ≥ 7.5 mmol/L, the risk of in-hospital mortality in hip fracture patients increased by 2.2-fold (43). Similarly, elevated creatinine levels (> 100 μmol/L) were associated with increased 30-day and 1-year mortality rates, with hazard ratios of 2.49 and 1.54, respectively (44). Moreover, β2-MG is a low-molecular-weight protein primarily produced by lymphocytes and other nucleated cells. Its clearance depends largely on the glomerular filtration rate, making it a sensitive marker of renal function. In this study, we found that each 1.0 mg/L increase in β2-MG levels was associated with a 4.2% increase in mortality risk, which has not been previously reported in the literature. In other diseases, elevated β2-MG levels have been shown to be associated with increased mortality risk, including cardiovascular and cerebrovascular diseases (coronary heart disease, stroke) (45), as well as chronic obstructive pulmonary disease (46).

Diabetes is highly prevalent among older adults, with a prevalence of 21.4% in our study. Hyperglycemia can induce osteoporosis and increase the risk of fragility fractures, while also lead to neuropathy and retinopathy, which may impair balance and gait, and thereby increase the risk of falls and hip fractures (47). Moreover, hyperglycemia can exacerbate inflammation, impair wound healing, and increase the risk of infections, all of which contribute to higher mortality rates. This study showed that for every 1.0 mmol/L increment in glucose, the risk of mortality increased by 3.7%. Consistent with this finding, previous studies have demonstrated that hip fracture patients with diabetes had a higher mortality risk compared to those without diabetes, regardless of the type of glycemic control therapy received (48).

Hip fracture patients often experience coagulation dysfunction due to traumatic stress, inflammatory response, and limited mobility (49). Coagulation abnormalities can manifest as hypercoagulable or hypocoagulable states. A hypercoagulable state increases the risk of thromboembolic events, such as deep vein thrombosis and pulmonary embolism, which contribute to morbidity and mortality. Conversely, a hypocoagulable state can lead to excessive bleeding, complicating surgical interventions and prolonging recovery. Both scenarios impair the body’s ability to manage the physiological stress of hip fracture and subsequent treatment, thereby increasing the risk of adverse outcomes. PT is a key indicator for assessing coagulation function. In the present study, each 1.0-second increase in PT was associated with a 15.1% elevation in the risk of short-term mortality. Asrian et al. (50) similarly reported that PT was a significant predictor of 1-year mortality in hip fracture patients. Additionally, other coagulation indicators, such as INR (17) and APTT (50), have been shown to be independently associated with mortality risk in these patients. It should be noted that our database lacked data on pre-admission anticoagulant therapy. Thus, future studies should explore the impact of pre-admission anticoagulant therapy on coagulation parameters among hip fracture patients to provide more conclusive evidences.

Based on clinical indicators such as age and CCI, this study further incorporated various blood indicators into the predictive models. The results showed significant enhancements in the models’ AUC, NRI and IDI, indicating that these blood indicators substantially improve the predictive performance of existing clinical models for assessing short-term mortality risk in hip fracture patients. As depicted in Figure 5, Model B achieved an AUC ranging from 0.820 to 0.826 after the inclusion of blood indicators selected by stepwise logistic regression, the Boruta algorithm, and Lasso regression. Similar findings were reported by Lu et al. (17), who developed a mortality prediction model for hip fracture patients using the Medical Information Mart for Intensive Care IV (MIMIC-IV) database. Their model incorporated one clinical feature (sepsis) and seven blood indicators (BUN, creatinine, MCH, MCV, INR, monocyte, neutrophil), attaining an AUC of 0.824 for predicting 30-day mortality. Another study constructed a nomogram model to predict postoperative mortality in hip fracture patients, integrating three blood indicators (albumin, sodium, hemoglobin) with age and CCI, and reported AUCs of 0.83, 0.79, and 0.77 for predicting mortality at 6 months, 1 year, and 3 years, respectively (37). Wang et al. (51) developed a predictive model for mortality following total hip arthroplasty in patients with femoral neck fracture. Their model incorporated three blood indicators (hemoglobin, creatinine, albumin) along with age, preoperative nutritional risk score, and ASA score, and obtained an AUC of 0.814.

This study has several strengths that contribute to its robustness and reliability. First, the continuous enrollment of patients over a period of more than 10 years minimizes selection bias and ensures a comprehensive representation of the patient population. This long-term enrollment period allows for a more accurate assessment of predictors of mortality in hip fracture patients. Second, we employed three distinct feature selection methods: stepwise logistic regression, the Boruta algorithm, and Lasso regression. This multi-method approach ensures a comprehensive and robust identification of predictors associated with 90-day mortality in hip fracture patients. Third, we included numerous clinical and blood indicators. This comprehensive approach allows for a thorough evaluation of the patients’ overall health status and mortality risk. By incorporating both clinical and biochemical data, we provide a more detailed understanding of the factors contributing to mortality in hip fracture patients.

However, this study also has certain limitations that should be acknowledged. First, the retrospective single-center design of this study inevitably restricts the external validity of the findings. Although cases were included consecutively, the potential for selection bias remains. Moreover, the data were derived from a single center in a predominantly Chinese population, which may limit the representativeness of the patient selection and the generalizability of the findings to other populations. Future multi-center prospective studies with more diverse and representative patient cohorts are needed to further validate our findings. Second, due to the limitations in data collection, some important variables were not available for analysis, such as the Abbreviated Mental Test Score (AMTS) in the NHFS, the Park Mobility Score in the AHFS. As a result, this study was unable to assess the added predictive value of blood indicators for these commonly used clinical models. Furthermore, potential missing data bias may have affected the results, as some important variables could not be obtained for all patients. Third, although the study encompassed a period of over 10 years, the sample size was relatively small, potentially limiting the statistical power of the analysis. Fourth, this study identified various blood indicators associated with 90-day mortality. However, the absence of a final predictive model limits the comprehensive evaluation of their predictive performance. Future studies should determine the optimal set of predictors to construct predictive models, incorporating calibration plot and the Hosmer-Lemeshow test to assess model fit. Additionally, the large number of variables identified may restrict their immediate applicability in clinical settings. Future studies should consider developing simplified models using fewer routinely available parameters to enhance their usability in daily practice.

## 5 Conclusion

This study identified various blood indicators associated with 90-day mortality in older patients with hip fracture. These indicators significantly enhanced the predictive ability of existing clinical models for short-term mortality risk. By utilizing these blood indicators, clinicians can comprehensively and objectively assess the physiological status of hip fracture patients at the time of admission, thereby facilitating the early identification of high-risk patients and guiding personalized treatment strategies.

## Data Availability

The raw data supporting the conclusions of this article will be made available by the authors, without undue reservation.
